# Health Care Utilization and Family Frustration: Do Health Insurance Coverage and Medical Homes for US Children Matter?

**DOI:** 10.1007/s10995-025-04127-1

**Published:** 2025-07-04

**Authors:** Gahssan Mehmood, Theodoros V. Giannouchos

**Affiliations:** 1 Department of Health Promotion, Education, and Behavior, Arnold School of Public Health, University of South Carolina, 915 Greene St, Columbia, SC 29208 USA; 2 Department of Health Policy and Organization, School of Public Health, The University of Alabama at Birmingham, 1665 University Blvd, Birmingham, AL 35233 USA

**Keywords:** Children, Healthcare utilization, Frustration, Health insurance, Medical home

## Abstract

**Objective:**

To examine the association of health services utilization and family frustration in getting health care services with the intersection of consistent and adequate health insurance coverage and access to medical home care among children in the US.

**Methods:**

We conducted a pooled, cross-sectional data analysis using the 2016–2023 National Survey of Children’s Health data. Survey-weighted multivariable logistic regressions were used to estimate the association of emergency department (ED) visits, preventive care utilization, and family frustration in getting services with the intersection of having consistent and adequate health insurance coverage and comprehensive access to medical home care.

**Results:**

Of 279,789 children representing 61.9 million children from 2016 to 2023, 79.0% had one or more preventive care visits, 17.9% had at least one ED visit, and 18.7% of families reported being frustrated in efforts to get care for their children in the past 12 months. Overall, 33.1% of children were consistently and adequately insured and had access to medical home care, while 21.0% had neither. Compared to children with consistent and adequate health insurance coverage and access to a medical home, children without either or both consistent and adequate health insurance coverage and access to medical home care were less likely to have at least one preventive care visit and, their families were more likely to report frustration in obtaining services.

**Conclusion for Practice:**

Improving access to medical home care is critical to minimize disparities in preventive care use and to improve health outcomes for children, beyond comprehensive health insurance coverage.

## Significance

Existing research highlights the importance of comprehensive health insurance coverage and access to medical home care for healthcare utilization in children. However, little is known about how the intersection of these factors influence healthcare access and utilization among children in the United States. This study contributes fresh insights by examining the association between health services utilization, family frustration in accessing care, and the intersection of insurance coverage and medical home access using the data from 2016 to 2023. By shedding light on these relationships using nationally representative data, our findings provide valuable evidence for policymakers and healthcare providers to develop targeted interventions aimed at reducing disparities in healthcare access and improving health outcomes in children.

## Introduction

Health insurance coverage is a key determinant of access to healthcare services for children in the United States (US) (DeVoe et al., 2009; Hoffman & Paradise, 2008; Newacheck et al., 1998). Limited or no out-of-pocket contributions for the provision of healthcare services can mitigate financial barriers and enhance access to care (Wisk et al., 2020; Yee et al., 2024). In turn, health insurance has been associated with the utilization of more healthcare services and improved health outcomes among children (Brantley & Ku, 2021; Levy & Meltzer, 2008; Quimbo et al., 2011). However, health insurance coverage does not guarantee access to and provision of needed healthcare services on its own (DeVoe et al., 2007), even though it makes healthcare more affordable overall (Bailey, 2022; Jost & Pollack, 2016).

Although only around 5% of children in the US are currently uninsured (Alker et al., 2020), estimates suggest around 34% of all children are underinsured (Yu et al., 2022), and these uninsured and underinsured children are facing challenges and barriers in accessing basic healthcare services (Redlener et al., 2016). Limitations and uncovered categories of services by health insurance providers can expose families with children to large out-of-pocket amounts, which often discourage seeking needed services (Kreider et al., 2016). Lack of health insurance coverage or underinsurance predisposes to delays or even foregoing preventive care (Cassedy et al., 2008; Flores et al., 2017) and increased reliance on emergency departments (ED), often as substitutes for primary care (American Academy of Pediatrics, 2004; Anyatonwu et al., 2022; Giannouchos et al., 2021; Montalbano et al., 2016; Neuman et al., 2014). In addition, social factors and stresses, as well as supply-side factors, further perpetuate difficulties in seeking needed care.

Even though having health insurance is an important contributor to the receipt of medical care, it is inadequate to promote care that is “accessible, family-centered, continuous, comprehensive, coordinated, compassionate, and culturally effective” (American Academy of Pediatrics, 2004, 2025; Arthur & Blewett, 2023) which has been described as the most comprehensive method of providing healthcare services to children and defined as a Medical Home Model (American Academy of Pediatrics, 2004, 2025). A Medical Home Model includes, but is not limited to, having a personal physician or nurse, having a usual source of care, not having trouble getting referrals, receiving family-centered care, and effective care coordination (The Child and Adolescent Health Measurement Initiative, 2009, 2019).

According to recent estimates, around 3% of children do not have a regular source of care, a key component of the Medical Home Model, and the number is significantly higher (around 21%) for children without insurance (Federal Interagency Forum on Child and Family Statistics, 2023). Numerous studies have shown a link between enhanced access to healthcare and having consistent health insurance coverage along with a usual source of care among children (Hoilette et al., 2009; Kreider et al., 2016; Smith et al., 2005; Starfield, 2000; Starfield & Shi, 2004). Hence, the ability to provide comprehensive care through having both health insurance coverage and access to a medical home can bolster access to routine health checks and preventive services, which may lessen the need for expensive acute-care services (Arthur & Blewett, 2023; McBurney et al., 2004). Despite the important contributions of previous studies, there is limited evidence on whether and how the interplay of health insurance coverage consistency and adequacy and access to the medical home is linked to healthcare service utilization and parental attitudes towards the receipt of services for children, using up-to-date data.

The objective of this study was to examine the association of health services utilization and family frustration in getting health care services with the intersection of consistent and adequate health insurance coverage and access to a medical home among children in the US, using 2016 to 2023 nationwide survey data. Providing nationwide, up-to-date evidence can aid policymakers in making informed decisions to improve access to care and health outcomes in children.

## Methods

We conducted a pooled, cross-sectional data analysis using the 2016 to 2023 National Survey of Children’s Health (NSCH) data (Ghandour et al., 2018; The Child and Adolescent Health Measurement Initiative, 2019). The NSCH is a publicly available, nationally representative survey that provides comprehensive data on all aspects of children’s health and well-being in the US. The survey is administered by the Maternal and Child Health Bureau of the Health Resources and Services Administration (HRSA MCHB), covers children from 0 to 17 years old, and collects data on a variety of aspects, such as physical and mental health, access to healthcare, family dynamics, neighborhood, and school environments. It uses a random sampling method to contact households with one or more children under the age of 18. More details about the survey methodology are available elsewhere (Boudreau et al., 2012; The Child and Adolescent Health Measurement Initiative, 2019).

### Dependent Variables

The dependent variables of interest in our study were family frustration in efforts to get health care services, ED visits, and preventive care utilization. Family frustration in efforts to get healthcare services was a categorical variable provided in the NSCH dataset as “never frustrated”, “sometimes frustrated,” and “usually or always frustrated”. The variable included in the analysis was dichotomized to indicate whether a family gets frustrated when they attempt to get services for children or not (never). ED visits were available in the data as a categorical variable with three categories (“none”, “visited ED one time” and “two or more visits”), which was converted to a dichotomous variable, indicating whether a child had at least one ED visit in the last 12 months or not. Finally, preventive care utilization was a dichotomous variable indicating whether children had received any preventive care services in the last 12 months or not.

### Main Independent Variable

Our main independent variable of interest was the intersection between consistency and adequacy of health insurance coverage and having access to a medical home. We constructed this composite variable using six variables that were available in the data (The Child and Adolescent Health Measurement Initiative, 2019). First, consistent and adequate health insurance coverage status was defined as a dichotomous variable indicating whether the child was consistently and adequately insured over the past 12 months or not, using the methodology developed by the Child and Adolescent Health Measurement Initiative (CAHMI) (The Child and Adolescent Health Measurement Initiative, 2009, 2019). The medical home access variable was constructed using a composite measure based on five components constructed from a total of 16 survey items. The composite measure aligned with the methodology developed by CAHMI, which indicated whether a child had a personal physician or nurse, a usual source of care, received family-centered care, had trouble getting referrals (access to referral), and received care coordination (The Child and Adolescent Health Measurement Initiative, 2009, 2019). We note that while the American Academy of Pediatrics (AAP) medical‑home definition has seven key components in total (American Academy of Pediatrics, 2004; Conners et al., 2017), the NSCH does not collect information on compassionate care or continuity of care (The Child and Adolescent Health Measurement Initiative, 2009), so these two aspects could not be incorporated. Children were considered to have access to a medical home if they satisfied the criteria for adequate care on the first three components, namely having a personal doctor or a nurse, having a usual source of care, and care that is family-centered (The Child and Adolescent Health Measurement Initiative, 2009, 2019). The final independent variable of interest was specified using the intersection of the two above-mentioned measures and resulted in four categories indicating whether a child was, (1) consistently and adequately insured with medical home access, (2) consistently and adequately insured but without medical home access, (3) inconsistently and inadequately insured but with medical home access, or (4) inconsistently and inadequately insured and without medical home access.

### Covariates

We included several demographic, socioeconomic, contextual, and health-related covariates to account for factors associated with healthcare utilization historically and based on data availability (Taylor & Salyakina, 2019). Demographic, socioeconomic, and contextual variables included age, sex, race/ethnicity, household primary language, family income (% of federal poverty level), out-of-pocket costs for childcare in the past 12 months, the child’s overall health status based on parent/guardian reports, whether the family avoided changing jobs to maintain health insurance, family resilience, supportive neighborhood, food insufficiency, coping with day-to-day demands of raising children, and the highest level of education of any adult in the household (The Child and Adolescent Health Measurement Initiative, 2019). We used the imputed income variable that was available in the data due to missing income information among some participants according to CAHMI guidelines.

### Statistical Analysis

Descriptive statistics were used to summarize the characteristics of the study sample, further stratified by the four different levels of health insurance consistency and adequacy, and medical home access. Comparisons to assess statistical significance across the four categories and the outcomes of interest were conducted using chi-squared tests. To explore the association of family frustration in efforts to get health care services for a child, ED visits, and preventive care services utilization, and the intersection between consistent and adequate health insurance coverage and medical home access, three survey-weighted multivariable logistic regressions were used, controlling for all covariates included in the descriptive analyses and survey-year fixed effects. We calculated the variance inflation factor (VIF) to assess multicollinearity. All VIF values were below 2, indicating that multicollinearity was not a concern in the regression models. We also conducted sensitivity analyses using only data from 2016 to 2019 and 2020 to 2023 separately, due to the pandemic, to assess the robustness of our findings, beyond the year-fixed effects used in our main analysis. All analyses were conducted using survey weights and were performed using Stata version 15 (Stata Corp, College Station, TX). The study used de-identified and publicly available data, and was deemed non-human subjects research by the University of [blinded for review] institutional review board.

## Results

### Descriptive Characteristics of all Children

Overall, our study included 279,789 children representing 61.9 million children aged 0 to 17 years from 2016 to 2023 (Table 1). Of those, 33.1% were consistently and adequately insured with medical home access, while 34.2% were consistently and adequately insured but without medical home access. Around 21.0% of children had neither consistent and adequate health insurance coverage nor had access to a medical home. A smaller percentage of children (11.8%) had access to medical homes but did not have consistent and adequate insurance coverage, and adequate insurance coverage.

**Table 1:** Descriptive characteristics overall and stratified by health insurance consistency & adequacy and medical home access among children 0–17 years of age from 2016 to 2023

	Total	Consistently and adequately insured with medical home access	Consistently and adequately insured but without medical home access	Inconsistently and inadequately insured but with medical home access	Inconsistently and inadequately insured and without medical home access	*P*
Weighted N	61,950,010	20,474,478	21,168,318	7,322,491	12,984,723	
%	100.0%	33.1%	34.2%	11.8%	21.0%	
ED Visit						< 0.001
No ED visit	82.1%	85.5%	80.0%	83.5%	79.4%	
One or more ED Visits	17.9%	14.5%	20.0%	16.4%	20.6%	
Preventive care utilization						< 0.001
No preventive care visit	21.0%	15.1%	23.5%	17.7%	28.0%	
One or more preventive care visits	79.0%	84.9%	76.4%	82.3%	72.0%	
Family frustrated when seeking care for children						< 0.001
Never frustrated	81.3%	92.1%	80.9%	84.2%	63.4%	
Family frustrated	18.7%	7.9%	19.1%	15.8%	36.6%	
Income status						< 0.001
0–99% of FPL	18.8%	13.3%	26.3%	9.4%	20.5%	
100-199% of FPL	20.9%	17.9%	25.2%	16.7%	21.1%	
200-399% of FPL	28.5%	28.0%	25.0%	33.9%	31.7%	
400% of FPL or greater	31.8%	40.7%	23.5%	39.9%	26.7%	
Race/Ethnicity of child						< 0.001
White	50.5%	59.1%	41.4%	62.1%	45.3%	
Black	12.6%	10.8%	16.0%	8.8%	12.1%	
Hispanic	25.9%	19.5%	31.1%	19.8%	31.2%	
Other	10.9%	10.6%	11.5%	9.4%	11.5%	
Age of child						< 0.001
0–3 years old	20.9%	22.9%	20.8%	21.2%	17.7%	
4–7 years old	21.8%	23.0%	22.0%	20.7%	20.1%	
8–11 years old	22.8%	22.2%	23.3%	21.9%	23.4%	
12–14 years old	17.5%	16.9%	17.2%	18.8%	18.1%	
15–17 years old	17.0%	15.0%	16.6%	17.3%	20.7%	
Sex of child						0.285
Female	49.0%	48.8%	48.8%	49.7%	49.3%	
Male	51.0%	51.2%	51.2%	50.3%	50.7%	
Child’s overall health status						< 0.001
Excellent	90.3%	94.7%	88.4%	93.0%	85.1%	
Good	8.2%	4.6%	9.8%	6.1%	12.3%	
Fair or Poor	1.5%	0.7%	1.8%	0.8%	2.6%	
Out of pocket cost for childcare in the past 12 months						< 0.001
$0 - $249	60.4%	67.5%	77.8%	27.2%	39.8%	
$250 - $499	14.5%	15.7%	10.4%	19.5%	16.4%	
$500 - $999	10.7%	8.9%	6.1%	20.4%	15.7%	
$1000 or more	14.4%	8.0%	5.8%	32.7%	28.1%	
Household language (primary)						< 0.001
English	85.2%	90.8%	81.0%	90.4%	79.9%	
Spanish/Other	14.9%	9.2%	18.9%	9.5%	20.2%	
Avoid changing jobs to maintain health insurance						< 0.001
No	93.3%	96.3%	95.7%	89.4%	86.6%	
Yes	6.8%	3.7%	4.3%	10.5%	13.5%	
Family resilience						< 0.001
More resilient family	83.1%	88.3%	81.5%	84.4%	76.9%	
Less resilient family	16.9%	11.7%	18.5%	15.6%	23.1%	
Supportive neighborhood						< 0.001
Yes	55.3%	64.4%	51.5%	59.1%	45.0%	
No	44.7%	35.6%	48.5%	40.9%	55.0%	
Highest level of education of any adult in the household						< 0.001
College degree or higher	51.2%	61.5%	39.7%	62.8%	47.1%	
Less than high school	8.9%	4.7%	12.2%	5.2%	12.2%	
High school degree or GED	18.8%	14.0%	25.1%	12.4%	19.6%	
Some college or technical school	21.1%	19.8%	22.9%	19.5%	21.2%	
Food insufficiency						< 0.001
Always enough nutritious food	68.5%	77.4%	64.6%	71.7%	59.3%	
Always enough food but not nutritious	26.1%	19.6%	28.9%	25.0%	32.7%	
Not enough food	5.3%	3.0%	6.6%	3.3%	8.0%	
Coping with the day-to-day demands of raising children						< 0.001
Very well	61.3%	67.0%	62.3%	59.1%	52.1%	
Somewhat well	36.9%	32.3%	35.9%	39.5%	44.4%	
Not very well or not very well at all	1.8%	0.8%	1.8%	1.4%	3.5%	

Most children (79.0%) had one or more preventive care visits in the past 12 months. About one in every six children (17.9%) had at least one ED visit in the past 12 months, while a similar share of families (18.7%) reported being frustrated in their efforts to get care for their children. Most families had incomes above 200% of the FPL (200-399% FPL = 28.5%; 400% or more FPL = 31.8%), a college degree or higher as the highest level of education of any adult in the household (51.2%), and identified as white (51.8%). The age and sex of the children were overall balanced. The majority of parents (90.3%) perceived their child’s health status as excellent, and most parents (68.5%) reported having always enough nutritious food, and 61.3% coped very well with the day-to-day demands of raising their children. About one in four parents reported out-of-pocket healthcare costs for their child’s care of $500 or more during the past 12 months ($500-$999 = 10.7%; $1000 or more = 14.4%), and about 6.8% reported avoiding changing jobs to maintain health insurance coverage.

### Stratified Descriptive Characteristics of Children by Health Insurance Consistency and Adequacy and Medical Home Access

**Fig. 1:**
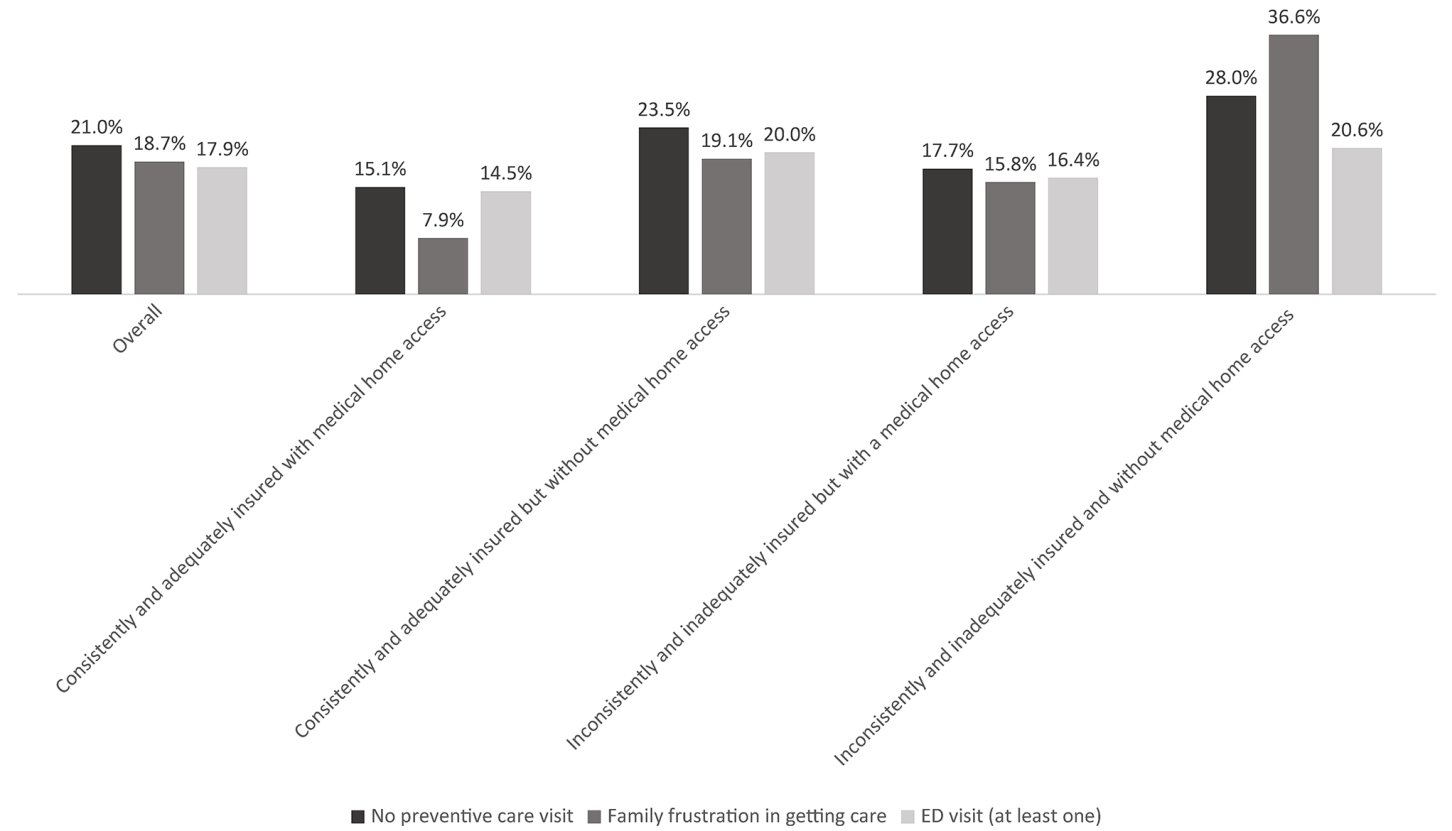
Comparison of preventive care utilization, family frustration, and ED visits stratified by health insurance adequacy and consistency and medical home access

Children without medical home access, independent of their consistent and adequate health insurance coverage status, were significantly more likely to have at least one ED visit over the past 12 months compared to those with both consistent and adequate health insurance coverage and access to medical home (p-value < 0.001) (Table 1; Fig. 1). In contrast, children with consistent and adequate insurance coverage and medical home access were disproportionately more likely to have at least one preventive care visit over the past 12 months (84.9%) compared to all other three groups, particularly compared to those with inconsistent and inadequate insurance and without medical home access (72.0%, p-value < 0.001). A similar association was observed for family frustration when seeking health care for children, with only 7.9% of parents of children with consistent and adequate insurance coverage and medical home access reporting frustration, compared to 36.6% of those with children without consistent and adequate insurance coverage and without medical home access (p-value < 0.001).

In general, compared to children with consistent and adequate insurance coverage and access to a medical home, all other groups that did not meet either or both criteria resided in households with lower income, were more likely to be Hispanic, to have a primary language other than English, and had lower adult educational levels. Similarly, children with inconsistent and inadequate health insurance coverage or those without medical home access or both were more likely to have worse health status, and less resilient family environments, while parents of these children were also more likely to report food insufficiencies and issues to cope with the day-to-day demands of raising children.

### Multivariable Regression Analyses

The results of multivariable logistic regressions are presented in Table 2. Compared to children with consistent and adequate health insurance coverage and medical home access, families of children with inconsistent and inadequate health insurance coverage and not having access to a medical home had more than four times higher odds of reporting frustration in obtaining health care services for their children (aOR: 4.37, 95% Confidence Intervals– CI: 4.06–4.69). A similar but smaller in magnitude association was also observed among those with inconsistent and inadequate coverage but with medical home access (aOR: 1.64, 95% CI: 1.50–1.81) and those without medical home access but consistent and adequate health insurance coverage (aOR: 2.33, 95% CI: 2.18–2.50). In contrast, the odds of having at least one preventive care visit during the past 12 months were significantly lower for children without either or both consistent and adequate health insurance coverage and medical home access compared to children with both of these characteristics. In particular, the lowest odds for preventive care visits were observed for children without both consistent and adequate health insurance coverage and medical home access (aOR: 0.44, 95% CI: 0.41–0.47). Finally, no significant association was observed between having at least one ED visit over the past 12 months and health insurance adequacy and consistency, and medical home access. Other covariates that were significantly associated with all three outcomes included having higher household income, family resilience, parental education, and food insufficiency. Of note, parental frustration with getting care for children was significantly and positively associated with not changing jobs to maintain health insurance coverage (aOR: 2.07, 95% CI: 1.93–2.23) and not coping well with day-to-day demands of raising children (aOR: 2.89, 95% CI: 2.44–3.42). Estimates from the sensitivity analyses using 2016 to 2019 and 2020 to 2023 data separately were similar, suggesting robust findings.

**Table 2:** Multivariable logistic regression estimates of the association of family frustration in efforts to get health care services for a child, ED visits, and preventive care services utilization, and the intersection between consistent and adequate health insurance coverage and medical home access

	Preventive care visits (at least one)	Family frustrated when seeking care for children	ED visits (at least one)
	AOR (95% CI)	AOR (95% CI)	AOR (95% CI)
Health insurance coverage and medical home environment comprehensiveness (Ref: consistently and adequately insured with medical home access)			
Consistently and adequately insured but without medical home access	0.73 (0.69, 0.78)	2.33 (2.18, 2.50)	1.20 (1.13, 1.27)
Inconsistently and inadequately insured but with a medical home access	0.65 (0.60, 0.71)	1.64 (1.50, 1.81)	0.92 (0.85, 1.00)
Inconsistently and inadequately insured and without medical home access	0.44 (0.41, 0.47)	4.37 (4.06, 4.69)	1.04 (0.97, 1.12)
Income status (Ref: 0–99% of FPL)			
100-199% of FPL	1.16 (1.07, 1.26)	0.94 (0.86, 1.03)	0.79 (0.73, 0.86)
200-399% of FPL	1.16 (1.07, 1.25)	0.76 (0.70, 0.83)	0.61 (0.57, 0.66)
400% of FPL or greater	1.48 (1.36, 1.60)	0.72 (0.66, 0.79)	0.62 (0.57, 0.67)
Race/Ethnicity of child (Ref: White)			
Black	0.98 (0.91, 1.05)	0.81 (0.74, 0.88)	1.54 (1.43, 1.66)
Hispanic	0.94 (0.88, 1.01)	1.01 (0.94, 1.08)	1.10 (1.03, 1.18)
Other	0.76 (0.72, 0.81)	0.93 (0.87, 1.00)	0.95 (0.89, 1.01)
Age of child (Ref: 0–3 years)			
4–7 years old	0.66 (0.60, 0.72)	1.13 (1.04, 1.22)	0.66 (0.62, 0.71)
8–11 years old	0.44 (0.41, 0.48)	1.17 (1.08, 1.26)	0.47 (0.44, 0.50)
12–14 years old	0.40 (0.37, 0.44)	1.10 (1.01, 1.20)	0.44 (0.41, 0.48)
15–17 years old	0.36 (0.33, 0.39)	1.14 (1.05, 1.24)	0.47 (0.44, 0.50)
Child sex (Ref: Female)			
Male	1.00 (0.96, 1.05)	1.04 (0.99, 1.09)	1.14 (1.08, 1.19)
Children’s overall health status (Ref: Excellent)			
Good	1.37 (1.24, 1.51)	2.22 (2.05, 2.40)	1.88 (1.73, 2.04)
Fair or Poor	1.62 (1.25, 2.11)	3.85 (3.20, 4.64)	3.06 (2.58, 3.64)
Out-of-pocket cost for childcare (Ref: Less than 250$)			
$250 - $499	1.37 (1.28, 1.47)	1.11 (1.04, 1.20)	1.15 (1.07, 1.23)
$500 - $999	1.61 (1.48, 1.75)	1.29 (1.20, 1.40)	1.57 (1.45, 1.70)
$1000 or more	1.84 (1.70, 1.99)	1.51 (1.41, 1.62)	2.20 (2.05, 2.36)
Household language - primary (Ref: English)			
Spanish/Other	0.75 (0.69, 0.82)	0.88 (0.79, 0.97)	0.85 (0.77, 0.94)
Avoid changing jobs to maintain health insurance (Ref: No)			
Yes	1.44 (1.30, 1.60)	2.07 (1.93, 2.23)	1.17 (1.08, 1.27)
Family resilience (Ref: More resilient family)			
Less resilient family	0.92 (0.86, 0.99)	1.25 (1.17, 1.33)	1.02 (0.95, 1.08)
Supportive neighborhood (Ref: Yes)			
No	1.12 (1.06, 1.18)	1.43 (1.35, 1.51)	1.10 (1.04, 1.16)
Highest level of education of any adult in the household (Ref: ≥College degree)			
Less than high school	0.42 (0.38, 0.48)	0.71 (0.62, 0.83)	1.42 (1.24, 1.62)
High school degree or GED	0.56 (0.52, 0.60)	0.77 (0.71, 0.82)	1.48 (1.38, 1.59)
Some college or technical school	0.68 (0.65, 0.73)	0.92 (0.87, 0.98)	1.46 (1.38, 1.55)
Food insufficiency (Ref: Always enough nutritious food)			
Always enough food but not nutritious	1.16 (1.10, 1.23)	1.49 (1.40, 1.58)	1.23 (1.16, 1.30)
Not enough food	1.11 (0.99, 1.25)	2.15 (1.92, 2.40)	1.49 (1.33, 1.67)
Coping with the day-to-day demands of raising children (Ref: Very Well)			
Somewhat well	1.14 (1.08, 1.20)	1.71 (1.62, 1.80)	1.00 (0.95, 1.05)
Not very well or not very well at all	1.02 (0.85, 1.22)	2.89 (2.44, 3.42)	1.15 (0.97, 1.36)

**Notes**: All estimates are weighted and included survey-year fixed effects. AOR: Adjusted Odds Ratios; CI: Confidence Intervals; Ref: Reference

## Discussion

Using nationally representative data from 2016 to 2023, we found that almost half of all children in the US did not have access to a medical home. We further documented that family frustration with seeking health care, and not using preventive healthcare services, was significantly associated with lack of access to a medical home and inconsistent and inadequate health insurance coverage. We did not document any significant variations in ED visits among children conditional on health insurance coverage and access to a medical home after controlling for covariates.

The findings of our study support the notion that consistent and adequate health insurance coverage alone does not guarantee access to and utilization of preventive care among children (DeVoe et al., 2012). In addition, the absence of key medical home components like a personal provider, a usual source of care, and receipt of family-centered care can also lead to increased parental frustration in their efforts to get services for their children, independent of the comprehensiveness of insurance coverage. Hence, using consistent and adequate health insurance coverage as a measure of access to care, without considering other contributors that are highly interrelated with health insurance such as medical home access, could be incomplete or misleading (DeVoe et al., 2008; Institute of Medicine et al., 2009; Lichstein et al., 2018; Raphael et al., 2009; Strickland et al., 2011). Our work extends previous literature by highlighting the importance of establishing relationships with providers in accessing and utilizing healthcare services, which can also alleviate the parental burden of seeking and getting care for their children.

Beyond consistent and adequate health insurance coverage and medical home access, we also documented long-standing socioeconomic disparities, which were particularly related to the receipt of preventive care. Children from lower-income and less educated families, as well as those reporting food insecurities and non-primary English language-speaking households, were less likely to have at least one preventive care visit in the past year, and parents from such families were more likely to report frustration in getting care for their children. These findings highlight that socioeconomic factors further amplify disparities and barriers to care, particularly among marginalized children who lack consistent and adequate health insurance and access to a medical home (Sasha et al. 2019; Stevens et al., 2010; Weller et al., 2020; Zickafoose & Davis, 2013).

With almost 20 million children in the US having issues obtaining healthcare services at a critical stage of their lives (Redlener et al., 2016), our findings highlight the positive impact of access to medical homes in promoting preventive services uptake and improving care-seeking experiences, using the most up-to-date available data. Although progress has been made in improving access to medical homes, over the years, there is still room for improvement (Stevens et al., 2010; Weller et al., 2020). The recent expansion of postpartum Medicaid coverage from 60 days to 12 months presents a policy development aimed at stabilizing maternal and children’s coverage (Clark & Burak, 2022; Johnson & Bruner, 2018). Recent evidence suggests that the expansion of postpartum Medicaid coverage has significantly increased health insurance access for new mothers and their children (Stone & Chandrasekaran, 2024). Consistent with the study’s findings, the benefits of prolonged coverage could potentially be strengthened by pairing the coverage extension with pediatric medical home access incentives, which could further promote preventive and primary care use (Clark & Burak, 2022). Additionally, findings of our study also suggest the need to align Medicaid payment reforms with the AAP medical home standards (Kusma et al., 2023), which may enhance care continuity for vulnerable pediatric populations and, in turn, contain ED use for primary care treatable or preventable conditions. Moreover, given the importance of medical home access in access to preventive services, as our findings suggest, states may be encouraged to report all the individual medical home components as additional performance metrics, enabling them to target resources to the specific areas that most need improvement, thereby enhancing access and outcomes for children (Centers for Medicare & Medicaid Services, n.d.). Finally, policymakers may incentivize providers’ and stakeholders’ outreach aimed at facilitating and building long-term relationships with children and their families, which can mitigate prevailing barriers that predispose negative experiences and barriers to obtaining preventive services beyond health insurance coverage. Combined efforts targeting children and their families holistically, beyond policies and measures focused on addressing barriers to care unidimensionally, are essential to effectively improve health outcomes and mitigate disparities.

### Limitations

This study is not without limitations. First, we used survey data based on parental self-reported information, which might be inaccurate due to response, recall, or social-desirability biases. Additionally, reliance on data collected from caregivers restricts the exploration of youth/children’s perspectives on their care, potentially introducing bias and overlooking crucial nuances in care experiences. Moreover, the study’s cross-sectional nature represents associations rather than causal relationships. Furthermore, despite efforts to mitigate bias through survey weights, the possibility of bias due to survey nonresponse remains, affecting the generalizability of results. Finally, while the survey components used to assess medical home status align closely with the definition from AAP (American Academy of Pediatrics, 2025), only five of the seven medical home domains were included in the definition of medical home due to data availability, which may bias our estimates.

## Conclusion

In conclusion, our study indicates that consistent and adequate health insurance coverage and having access to a medical home can improve parental experiences with getting healthcare services for their children and improve the provision of preventive services at a critical developmental age. Improving access to medical homes is critical to minimize disparities in access to preventive care and to improve health outcomes for children, beyond health insurance coverage.

## Data Availability

The data used to generate the results is available upon request from the corresponding author.

## Code Availability

The code used to generate the results is available upon request from the corresponding author.
